# *CO*_2_*FE*: A Novel Metric for the Carbon Footprint Efficiency of Standard Laboratory Chemicals in Life Science Research

**DOI:** 10.3390/mps9050143

**Published:** 2026-10-02

**Authors:** Bero H. G. Schnell, Selina Lang, Jonas Follmann, Dirk Holtmann

**Affiliations:** Process Engineering in Life Sciences 2—Electro Biotechnology, Karlsruhe Institute of Technology, 76131 Karlsruhe, Germany; bero.schnell@kit.edu (B.H.G.S.); selina.lang@kit.edu (S.L.);

**Keywords:** carbon footprint, sustainable laboratory work, quantitative assessment, *CO*_2_ *FE*

## Abstract

Industrial activities and the transportation sector are widely recognized as two of the main contributors to global carbon dioxide (CO_2_) emissions. Nevertheless, research laboratories likewise consume significant quantities of energy and consumables, resulting in a substantial environmental impact. Life-cycle assessment (LCA) studies of individual reagents and openly accessible databases make it possible to quantify the carbon footprint of compounds and processes. These data allow researchers to compare alternatives and deliberately choose chemicals with a smaller carbon footprint, promoting more sustainable experimental practices. As LCA is often time-consuming and data-intensive, a simpler parameter is required. The novel parameter CO_2_ footprint efficiency (*CO*_2_*FE*) allows for a deliberate, greener choice to minimize emissions without compromising experimental performance. The proposed method comprises four steps. First, alternative reagents are identified from the literature, protocols or lab experience. Second, if possible, each option is assessed using data that account for material, production and life-cycle. Third, the most promising candidates are experimentally validated to ensure that sustainability gains do not compromise yield, activity, or reproducibility. Finally, the *CO*_2_*FE* is calculated to quantify carbon-efficiency. A lower *CO*_2_*FE* indicates a greener experiment. By iterating this workflow, researchers can systematically choose chemicals that reduce the overall carbon footprint without sacrificing scientific quality.

## 1. Introduction

While industry and transportation are among the largest sources of CO_2_ emissions, research laboratories also consume significant amounts of energy, materials, and consumables, contributing to their environmental footprint. Reducing the environmental impact of laboratory activities requires reliable methods to assess the sustainability of experimental procedures [1]. Furthermore, the choice of chemicals in the laboratory is often based on historical experiments rather than knowledge of the impact of applied chemicals on the environment. This makes it necessary to rethink laboratory work and to take a more sustainable approach to minimize the environmental impact of research. Chemicals, which are produced by the global chemical industry, which contributes around 5% of global CO_2_ emissions [2], are a major consumable for many research fields, especially those related to chemistry and biotechnology. Life-cycle assessments (LCAs) of chemicals [3] and the accessibility of these data in environmental databases such as ecoinvent.org [4,5] enable the carbon footprint of individual chemicals to be evaluated, creating the possibility of raising awareness of these chemicals and making a comparable selection. LCA is widely recognized as a robust tool for quantifying the environmental impact of products and processes throughout their entire life-cycle. However, conducting a full LCA often requires substantial amounts of data, expertise and computational effort. This can make implementation costly and time-consuming, particularly during the early design stages or when multiple alternatives must be evaluated. Consequently, it is desirable to have a simplified parameter that enables faster decision-making while still capturing the most relevant environmental aspects.

Adopting a carbon footprint-driven approach to selecting standard laboratory chemicals reveals the environmental impact of common choices in experimental design, e.g., regarding buffer systems for enzyme catalysis, pH control during microbial cultivation, and carbon source selection. Buffers are essential for maintaining optimal activity and stability in enzymatic reactions. However, commonly used buffering agents differ in their life-cycle emissions depending on how they are synthesized, purified, and disposed of. For example, phosphate buffers are widely recognized for their biochemical compatibility; however, the source of the phosphate can differ in its carbon footprint [6,7]. This suggests that careful evaluation of performance and sustainability is necessary. Similarly, adjusting pH during cultivation often relies on strong acids and bases, such as hydrochloric acid or potassium hydroxide. The industrial production of these chemicals is energy-intensive [8,9]. Alternative strategies, such as using buffering media or weaker acids and bases, can reduce the need for repeated titration and lower overall emissions. The choice of carbon sources in cultivations also plays a critical role because substrates can vary significantly in their environmental impact depending on their origin (e.g., petrochemical versus renewable biomass) and processing requirements [10,11,12].

Overall, integrating carbon footprint considerations into the selection of standard laboratory chemicals encourages a shift towards more sustainable experimental practices, balancing efficiency, availability, and environmental responsibility to minimize the environmental impact of routine laboratory work. One strategy for establishing more sustainable experimental designs could be to calculate the here-introduced CO_2_ footprint-based efficiency (*CO*_2_*FE*) of experiments, taking into account the carbon footprint of the chemicals used against an experimental output parameter.

## 2. Method Details

**Scheme 1:**
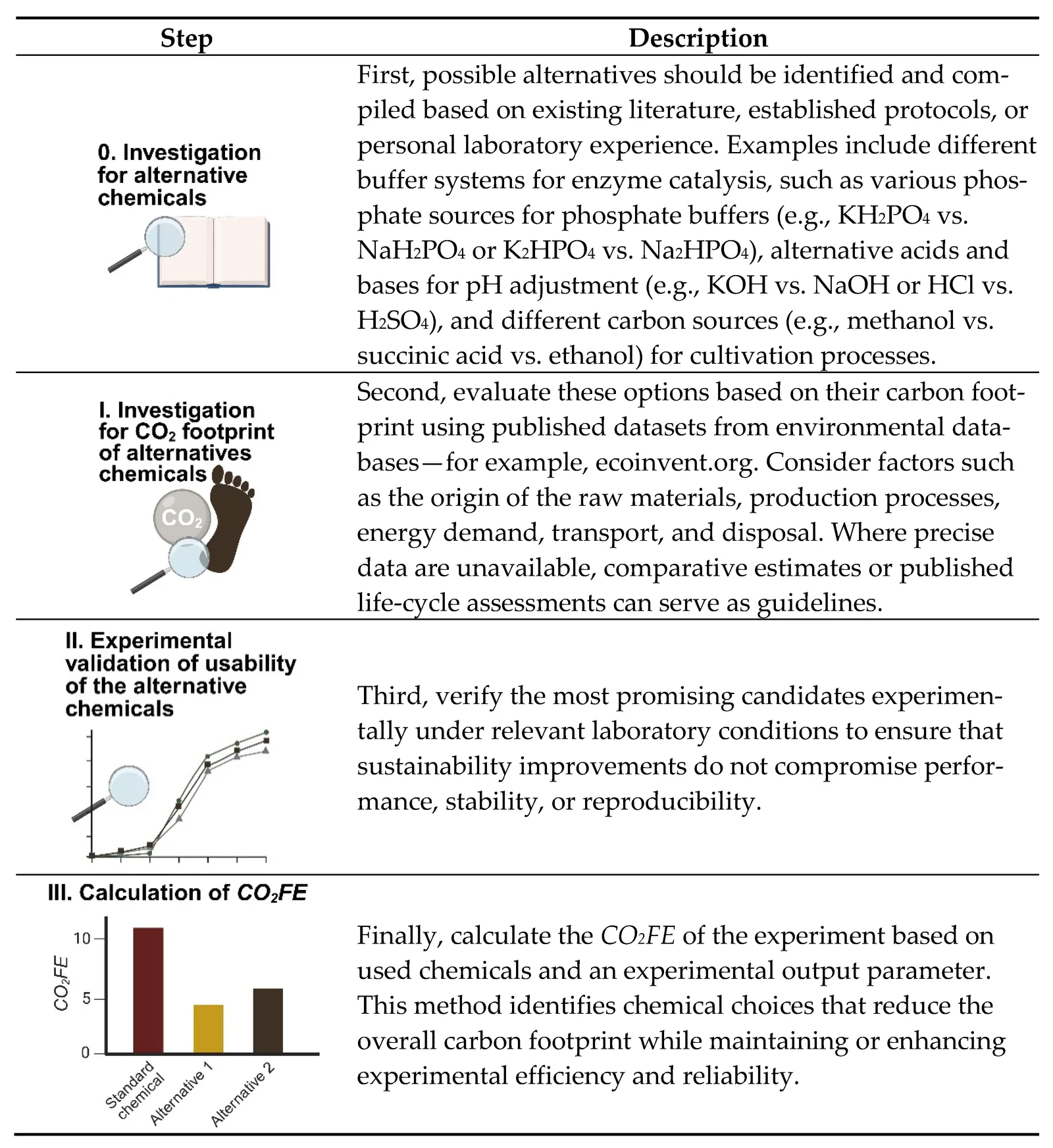
Overview of the carbon footprint-driven approach to selecting standard laboratory chemicals.

The general method for implementing a carbon footprint-driven approach to selecting standard laboratory chemicals can be broken down into the four main steps shown in Scheme 1.

The suitable key performance indicator (KPI) for this approach is a here-introduced CO_2_-based efficiency metric, the *CO*_2_*FE*, which relates the carbon footprint of chemicals to different experimental output parameters. This experimental output parameter is not a single predefined variable but rather an application-specific performance indicator that reflects the desired outcome of an experiment. Depending on the laboratory application, this can include parameters such as volumetric enzymatic activity, biomass concentration, product yield, product titer, reaction rate, or other relevant key performance indicators. Therefore, the *CO*_2_*FE* can be described as “experiment-related chemical CO_2_ footprint per functional unit”, where the functional unit represents the desired laboratory outcome. The metric can be expressed as follows:

$${CO}_{2}FE=\;\frac{\sum_{k=1}^{n}{{exCO}_{2}}_{k}}{experimental\;output\;parameter}$$

where *CO*_2_*FE* is the CO_2_ footprint efficiency in [mg/x]. The numerator of the fraction represents the total CO_2_ footprint of the experiment (*exCO*_2_), while the denominator *experimental output parameter* represents the target value [x]. The unit depends on the investigated experimental output parameter. The purpose of *CO*_2_*FE* is to relate the carbon footprint of the applied chemicals to the specific experimental objective, thereby enabling meaningful comparisons across different laboratory workflows.

This *CO*_2_*FE* allows for the direct comparison of different chemicals by considering the amount of carbon emissions needed to achieve the same result. A lower *CO*_2_*FE* value indicates a more sustainable and efficient system. Importantly, this metric regards environmental and performance factors, ensuring that reductions in the carbon footprint do not compromise the effectiveness of experiments. The *CO*_2_*FE* can be adapted for specific applications, such as “CO_2_-eq per volumetric enzymatic activity” or “CO_2_-eq per biomass”. This makes the *CO_2_FE* a flexible and scalable target variable for more environmentally friendly laboratory practices. The *CO*_2_*FE* calculation does not reflect an experiment’s overall CO_2_ footprint; it compares several options while excluding all other parameters. This is possible if the other parameters are constant (e.g., energy demand, amount of used consumables (see examples below)).

## 3. Method Evaluation

Several stages of laboratory work offer opportunities to replace chemicals with greener alternatives, thereby enhancing overall sustainability. Examples include the buffers used for enzymatic reactions, the pH-control systems employed in microbiological cultivations and the selection of carbon sources. Here we present the *CO*_2_*FE* for these three application areas, demonstrating how a more sustainable laboratory workflow with greener choices can be achieved without any loss in performance. All laboratory procedures, database data and calculations can be found in the Supplementary Material.

### 3.1. Example 1: Determining the CO_2_FE of Different Phosphate Buffers for Enzymatic Assays

**Scheme 2:**
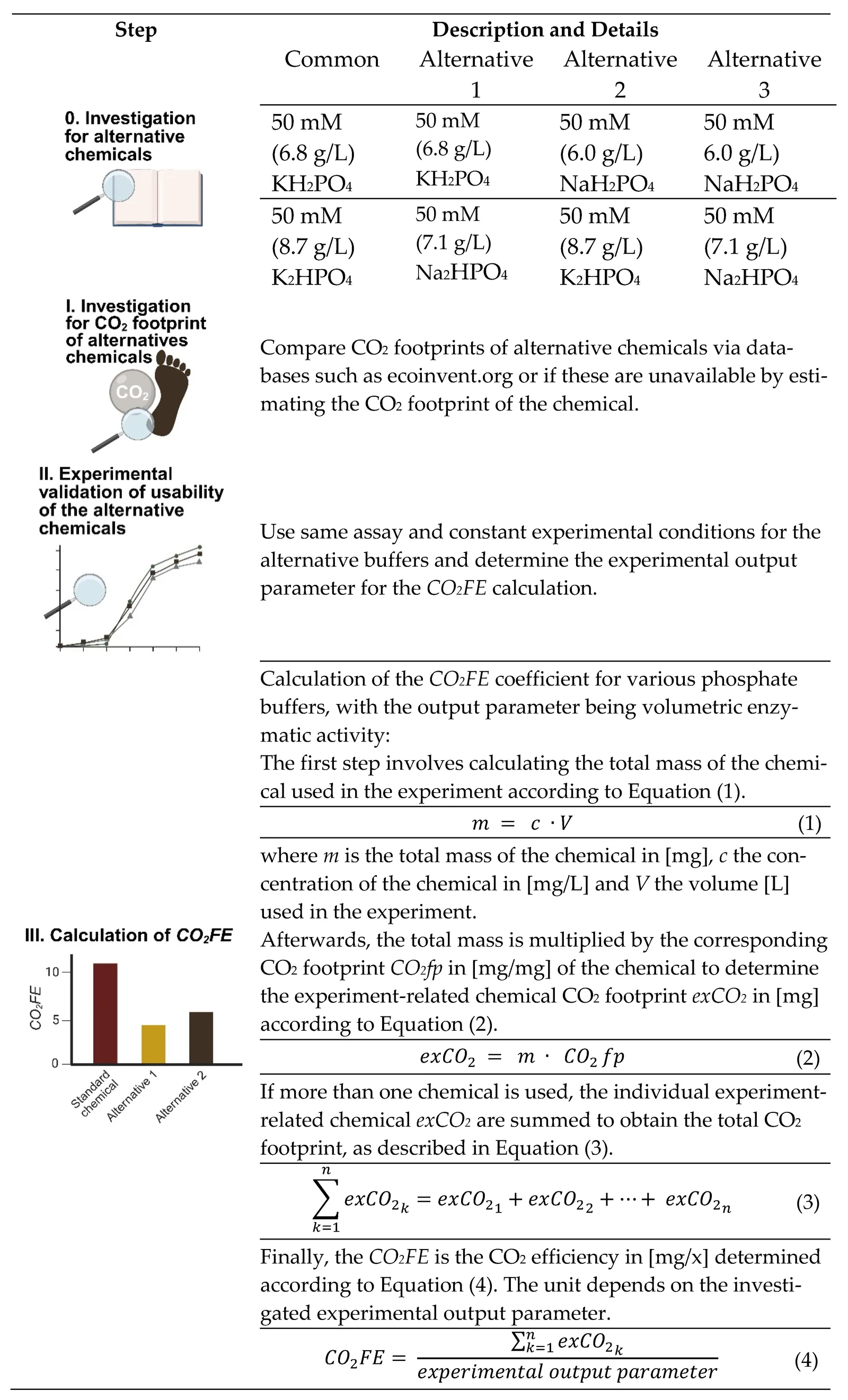
Determination of the *CO*_2_*FE* of different phosphate buffers for enzymatic assays.

**Figure 1:**
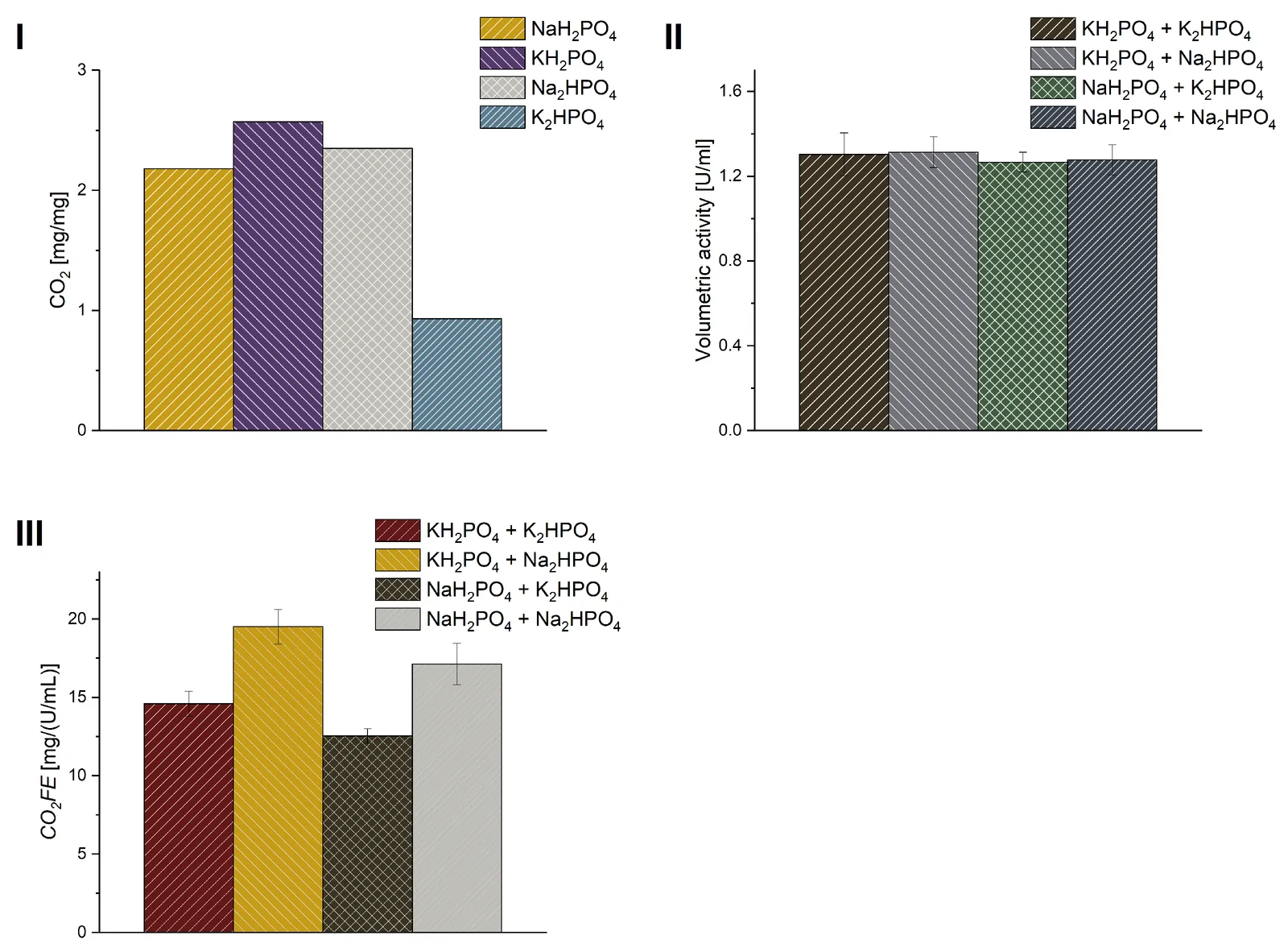
Validation of the *CO*_2_*FE* calculation for four different buffer compositions. The CO_2_ footprint of individual buffer components [6,7] taken from ecoinvent.org v3.12 https://ecoquery.ecoinvent.org/3.12/cutoff (accessed on 12 Janurary 2026) or estimated (see Supplementary Material) (**I**); the volumetric activity of *Aae*UPO obtained in the NBD assay, which serves as the experimental parameter investigated for the *CO*_2_*FE* and is reported as the mean ± standard deviation (*n* = 3) (**II**); and the calculated *CO*_2_*FE* values for the four buffer combinations, including propagated uncertainties (**III**).

Enzymatic reactions require a reliable buffer system to ensure reproducible measurements for optical activity and stability. Although a mixture of KH_2_PO_4_ and K_2_HPO_4_ is commonly employed, our investigation of the CO_2_ footprint of various buffer components [6,7]—combined with performance testing the buffers using the enzyme *Agrocybe aegerita* unspecific peroxidase (*Aae*UPO)—showed that all four tested buffer compositions yielded comparable enzymatic activity (Figure 1I,II). The methodology for determining the *CO*_2_*FE* of different 100 mM phosphate buffers, based on the volumetric enzymatic activity of *Aae*UPO in a 1,2-(Methylenedioxy)-4-nitrobenzene (NBD) assay [13], is demonstrated in the following section and shown in Scheme 2 step by step (the experimental details can be found in the Supplementary Material).

When the CO_2_ footprint is taken into account, the *CO_2_FE* can be calculated with Equation (4). This analysis reveals that, in terms of *CO*_2_*FE*, the conventional KH_2_PO_4_/K_2_HPO_4_ buffer is inferior to the NaH_2_PO_4_/K_2_HPO_4_ combination, which exhibits the best efficiency (Figure 1III). By using the ‘right’ buffer, the carbon footprint can be reduced by a factor of 1.3 without compromising the performance of the experiment.

### 3.2. Example 2: Determining the CO_2_FE of Different pH Control Systems in Fermentation Processes

**Figure 2:**
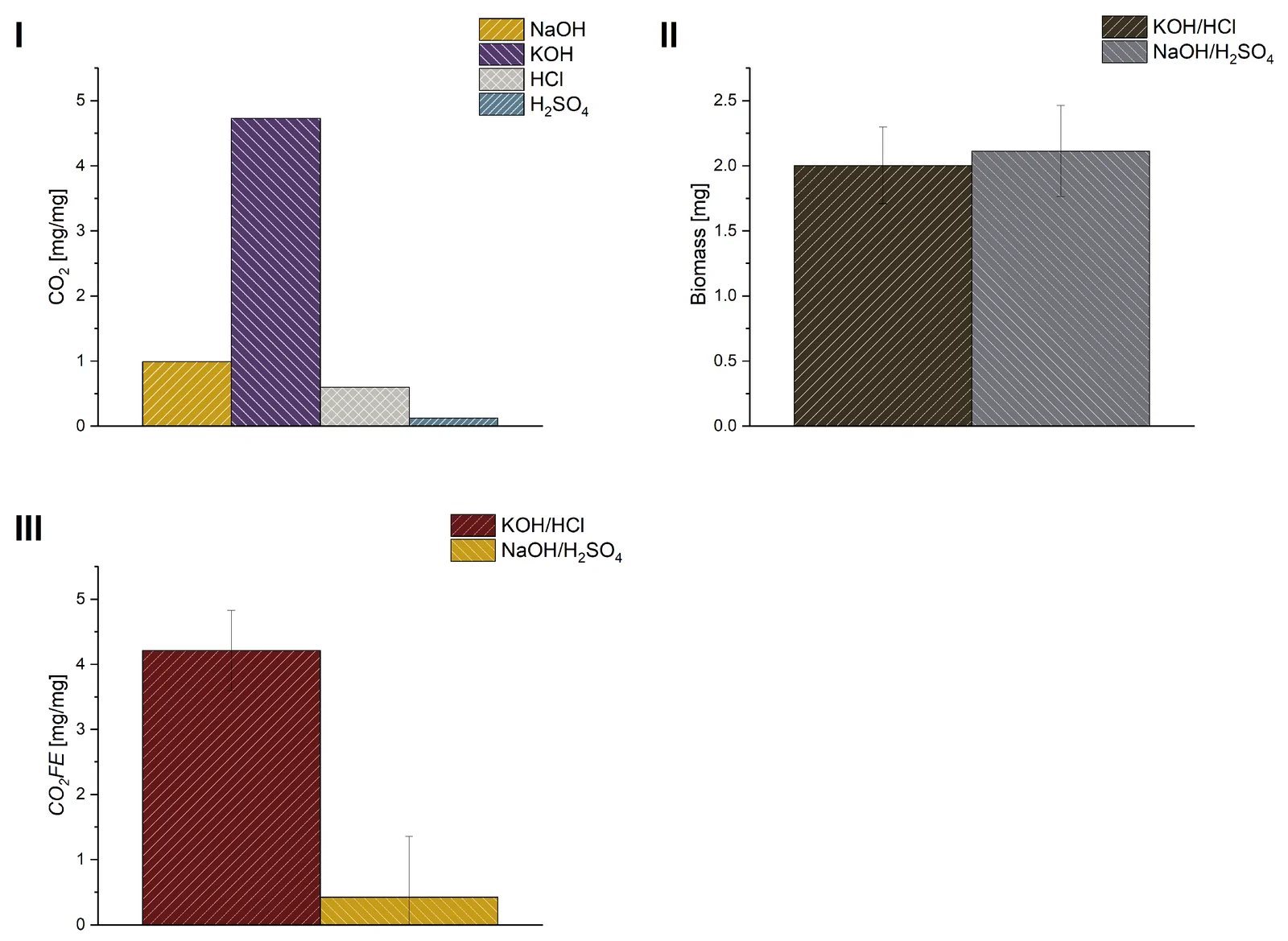
Validation of the *CO*_2_*FE* calculation for two pH correction systems. The CO_2_ footprint of the pH correction agents [8,9,14,15] taken from ecoinvent.org v3.12 https://ecoquery.ecoinvent.org/3.12/cutoff (accessed on 12 Janurary 2026) (**I**); the biomass of *V. natriegens* obtained through cultivation in a BioLector XT^®^ (Beckmann Coulter, Brea, CA, USA), which serves as the experimental parameter investigated in this study and is reported as the mean ± standard deviation (*n* = 3) (**II**); and the calculated *CO*_2_*FE* values for the two pH systems, including propagated uncertainties (**III**).

Controlling pH with appropriate correction agents is essential for reliable cultivations. After evaluating the CO_2_ footprint of promising alternatives (see Figure 2I) and growing *Vibrio natriegens* under pH-controlled conditions, the investigated parameter biomass is as presented in Figure 2II. The data show that all tested systems produce highly comparable amounts of biomass. When the CO_2_ footprint of the chemicals used is taken into account, the *CO*_2_*FE* plotted in Figure 2III clearly recommends using NaOH/H_2_SO_4_ for pH regulation instead of KOH/HCl. As in Example 1, using a chemical with a smaller carbon footprint enables the experiment’s performance to be maintained while reducing its carbon footprint by 89%.

### 3.3. Example 3: Determining the CO_2_FE of Different Carbon Sources

**Figure 3:**
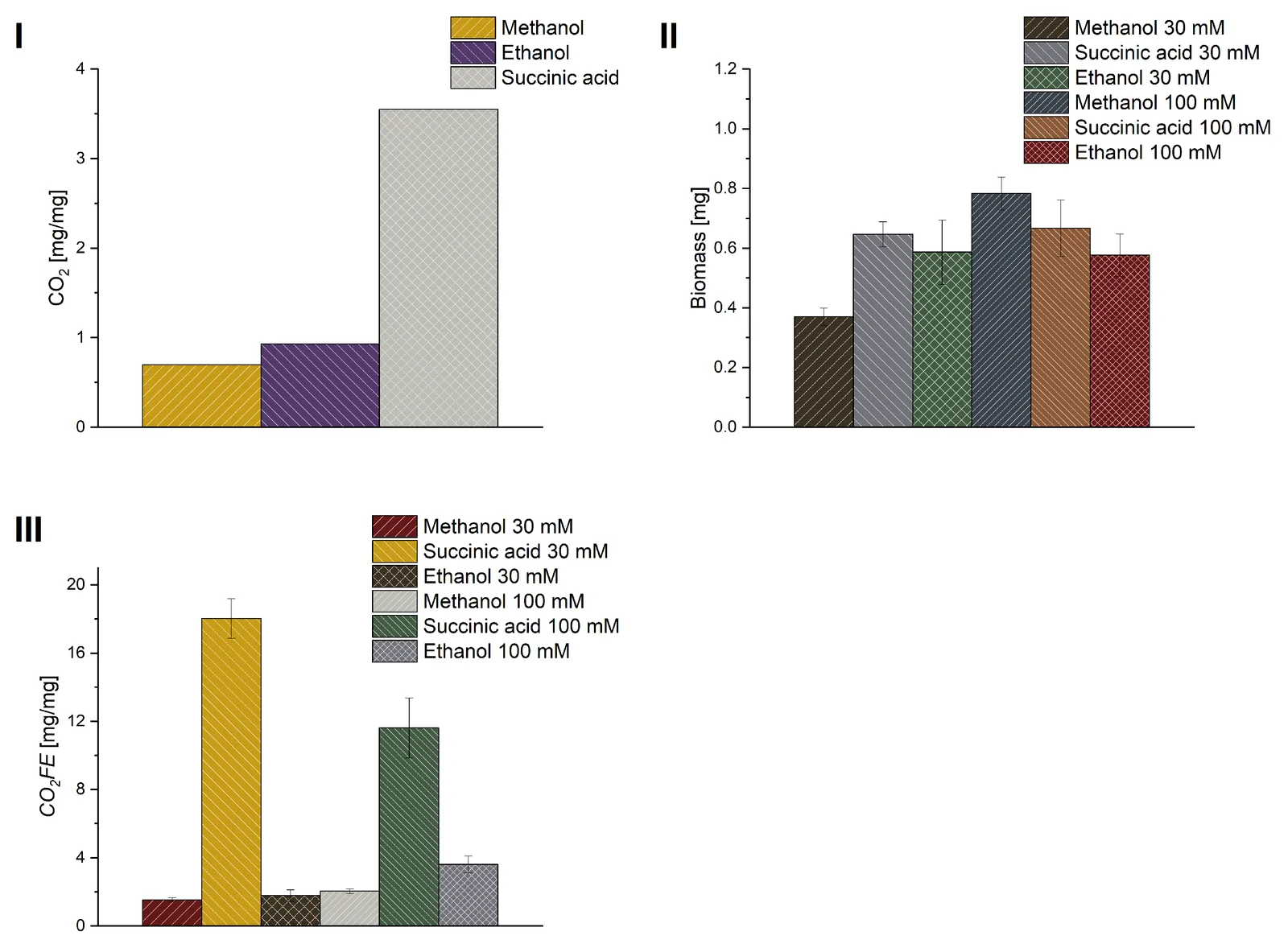
Validation of the *CO*_2_*FE* calculation for three carbon sources, each tested at two concentrations. The CO_2_ footprint of the carbon sources [10,11,12] taken from ecoinvent.org v3.12 https://ecoquery.ecoinvent.org/3.12/cutoff (accessed on 12 Janurary 2026) (**I**); the biomass of *M. extorquens* obtained during BioLector XT^®^ (Beckmann Coulter, Brea, CA, USA) cultivation, which is the experimental parameter under investigation for the *CO*_2_*FE* and is reported as the mean ± standard deviation (*n* = 3) (**II**); and the calculated *CO*_2_*FE* values for the carbon sources, including propagated uncertainties (**III**).

For the third case study, we investigated how different carbon sources affect biomass generation. The facultative methylotroph *Methylorubrum extorquens* can utilize methanol, succinic acid and ethanol as carbon substrates. The organism was cultured at two concentrations of each carbon source, and the amount of biomass generated was measured relative to the CO_2_ footprint of the substrate consumed (Figure 3I,II). In both concentration ranges, ethanol and methanol outperformed the commonly employed carbon sources succinic acid concerning the *CO*_2_*FE*, which yields the highest biomass per unit of CO_2_ emitted (Figure 3III). Unlike the previous examples, there are significant differences in the output parameters here. Nevertheless, determining *CO*_2_*FE* enables a comparison to be made based on the CO_2_ footprint relative to biomass. This demonstrates the parameter’s broad applicability.

## 4. Conclusions and Limitations

The main limitation of this approach is the availability of reliable CO_2_ footprint data for the chemicals applied. Many compounds, particularly trace elements and ionic substances, are not represented in commonly used life-cycle inventory databases such as ecoinvent.org [4] or CarbonCloud.com [16]. Furthermore, even some widely utilized chemicals may lack corresponding datasets, which can limit the completeness and accuracy of the environmental assessment. In such cases, the CO_2_ footprint must be estimated using proxy data or by selecting datasets for chemically similar compounds, a practice that is commonly applied in LCA and life-cycle impact assessment studies when substance-specific inventory data are unavailable [17,18] The uncertainty associated with emission factors can have a significant impact on absolute CO_2_ footprints in life-cycle assessments—particularly where data is scarce or poorly documented. However, this uncertainty often does not alter the ranking of the alternatives under consideration, as the relative differences between the options remain robust. This reduces the accuracy of the *CO*_2_*FE*. Furthermore, even when some chemicals are available in databases, the data can vary regarding the boundaries of their CO_2_ footprint assessment. For example, a distinction exists between transforming activities, which account solely for the production process, and market activities, which additionally include the distribution logistics of the respective chemicals (Supplementary Table S1). Consequently, the assessment boundaries of chemicals within an experiment may deviate, which limits the comparability of the CO_2_ footprint. Additionally, this parameter only considers the CO_2_ footprint of addressed chemicals within an experiment. The total CO_2_ footprint and respective *CO*_2_*FE* do not include the CO_2_ footprint of water, consumables such as plastic waste, or energy consumption. Another limitation is the lack of transparency among chemical distributors regarding the CO_2_ footprint of their products. This implies that, when a chemical is purchased from a distributor, the underlying production pathway often remains unclear, including whether the distributor produced the chemical, the origin of the supplied material, and which raw materials were used. However, these factors substantially influence the CO_2_ footprints reported in the available datasets. Moreover, when calculating the experiment-related chemical CO_2_ footprint for bacterial carbon sources, the mass of the carbon source has to be defined carefully. One approach is to consider the actual amount consumed by the organism, which reflects the true resource consumption. An alternative is to count the total amount added to the medium, because after cultivation the residual carbon is not recovered and therefore represents a waste stream. Relying solely on the added mass can be misleading if an excess of substrate is supplied, obscuring the organism’s genuine uptake efficiency. Consequently, both perspectives—(i) the carbon introduced into the medium and (ii) the carbon actually metabolized by the organism—are valid, but they yield different interpretations of the *CO*_2_*FE* and should be reported transparently.

The novelty of the proposed *CO*_2_*FE* metric does not lie in generating new carbon-footprint data, but in providing a standardized laboratory-oriented performance indicator that combines environmental impact and experimental outcome within a single quantitative measure. Existing carbon-footprint metrics typically report emissions per unit mass of product or chemical, whereas *CO*_2_*FE* relates experiment-specific carbon emissions directly to a functional laboratory output such as enzymatic activity, biomass formation, or product yield. This enables researchers to compare alternative laboratory chemicals while simultaneously considering sustainability and experimental performance within a single framework.

## Data Availability

The data presented in this study are available on request from the corresponding author.

## Supplementary Materials

The following supporting information can be downloaded at: https://www.mdpi.com/article/10.3390/mps9050143/s1. Table S1: Overview of CO_2_; footprints categorized by activity type, geographical activity origin (GLO: global; RER: Europe; EU: European Union; RoW: Rest of World), and database source; Table S2: Shows the different phosphate buffer alternatives with their respective concentrations used for the enzymatic assays; Table S3: Lists the components of the NBD enzyme assay and their respective concentrations; Table S4: Lists the molecular masses, the CO_2_; footprints obtained from ecoinvent.org v3.12 https://ecoquery.ecoinvent.org/3.12/cutoff (accessed January 12 2026), and the estimated CO_2_; footprints of the compounds included in the reaction equation; Table S5: Lists the molecular masses, the CO_2_; footprints obtained from ecoinvent.org v.3.12 https://ecoquery.ecoinvent.org/3.12/cutoff (accessed on 12 January 2026), and the estimated CO_2_; footprints of the compounds included in the reaction equation; Table S6: Composition of minimal medium and LBvn medium for *Vibrio natriegens* cultivation; Figure S1: Shows the BioLector XT^®^ (Beckmann Coulter, Brea, CA, USA) cultivation profiles of *V. natriegens* grown in minimal medium containing 10 g/L glucose over 48 h, including all monitored channels (black: backscatter; green: pH; red: dissolved oxygen). Data points represent cultures with KOH/HCl-based pH regulation, while crosses indicate cultures regulated using NaOH/H_2_SO_4_; Table S7: Lists the different components and their final concentrations in the minimal medium for *Methylorubrum extorquens*.
