# Supplementary material for: CO2FE: A Novel Metric for the Carbon Footprint Efficiency of Standard Laboratory Chemicals in Life Science Research
Source: Methods Protoc. 2026 Oct 2;9(5):143. doi: 10.3390/mps9050143 (PMC13707458; doi:10.3390/mps9050143)
Supplement: Supplementary file 1 [file mps-09-00143-s001.zip › mps-4581203-supplementary.pdf]

# *$CO_2FE$: A* Novel Metric for the Carbon Footprint Efficiency of Standard Laboratory Chemicals in Life Science Research

**Supplementary Material**

The $CO_2$ footprint were assessed via ecoinvent.org. In Supplementary Table S1 the dataset type and geographical assessment of data are represented.

Supplementary Table S1. Overview of $CO_2$ footprints categorized by activity type, geographical activity origin (GLO: global; RER: Europe; EU: European Union; RoW: Rest of World), and database source.

| Chemical | $CO_2$ footprint | Activity type | Geographic origin | Database | Dataset |
|---|---|---|---|---|---|
| $KH_2PO_4$ | 2.57 kg/eq | Estimated, see below | - | - | |
| $K_2HPO_4$ | 0.93 kg/eq | Estimated, see below | - | - | |
| $NaH_2PO_4$ | 2.18 kg/eq | Market activity | GLO | ecoquery.ecoinvent.org/3.12/cutoff | [7] |
| $Na_2HPO_4$ | 2.35 kg/eq | Market activity | GLO | ecoquery.ecoinvent.org/3.12/cutoff | [6] |
| KOH | 4.73 kg/eq | Market activity | GLO | ecoquery.ecoinvent.org/3.12/cutoff | [9] |
| NaOH | 0.99 kg/eq | Market activity | RER | ecoquery.ecoinvent.org/3.12/cutoff | [14] |
| HCl | 0.60 kg/eq | Market activity | RER | ecoquery.ecoinvent.org/3.12/cutoff | [8] |
| $H_2SO_4$ | 0.12 kg/eq | Market activity | RER | ecoquery.ecoinvent.org/3.12/cutoff | [15] |
| Methanol | 0.71 kg/eq | Market activity | RER (w.o. Russia) | ecoquery.ecoinvent.org/3.12/cutoff | [10] |
| Ethanol | 0.93 kg/eq | Market activity | RoW | ecoquery.ecoinvent.org/3.12/cutoff | [12] |
| Succinic acid | 3.55 kg/eq | Market activity | GLO | ecoquery.ecoinvent.org/3.12/cutoff | [11] |

**Enzymatic assay**

To test the volumetric enzymatic activity of the non-specific peroxygenase (*Aae*UPO) of *Agrocybe aegerita*, different phosphate buffers at a concentration of 100 mM were used (see Supplementary Table S2).

Supplementary Table S2. Shows the different phosphate buffer alternatives with their respective concentrations used for the enzymatic assays.

| Common | Alternative 1 | Alternative 2 | Alternative 3 |
|---|---|---|---|
| 50 mM (6.8 g/L) $KH_2PO_4$ (Carl Roth, Germany) | 50 mM (6.8 g/L) $KH_2PO_4$ (Carl Roth, Germany) | 50 mM (6.0 g/L) $NaH_2PO_4$ (Carl Roth, Germany) | 50 mM (6.0 g/L) $NaH_2PO_4$ (Carl Roth, Germany) |
| 50 mM (8.7 g/L) $K_2HPO_4$ (Carl Roth, Germany) | 50 mM (7.1 g/L) $Na_2HPO_4$ (Carl Roth, Germany) | 50 mM (8.7 g/L) $K_2HPO_4$ (Carl Roth, Germany) | 50 mM (7.1 g/L) $Na_2HPO_4$ (Carl Roth, Germany) |

To determine the enzymatic activity, a 1,2-(Methylenedioxy)-4-nitrobenzene (Sigma-Aldrich, MO, USA) (NBD) assay was performed, measuring the increase in 4-nitrocatechol absorption at 425 nm over 1.5 minutes [14]. The composition of the assay is shown in Supplementary Table S3, and the assay was performed in 1.5 mL cuvettes at 25 °C. The components were pipetted as described in Supplementary Table S3. The solution was blanked before $H_2O_2$ (Carl Roth, Germany) was added. All samples were measured in triplicates.

Supplementary Table S3. Lists the components of the NBD enzyme assay and their respective concentrations.

| Component | Concentration of stock solution | Volume in assay | Order of pipetting |
|---|---|---|---|
| Phosphate buffer (see Table S2) | 100 mM | 775 µL | 1 |
| NBD(dissolved in acetonitrile) | 5 mM | 100 µL | 2 |
| *Aae*UPO | n.i. | 100 µL | 3 |
| $H_2O_2$ | 40 mM | 25 µL | 4 |

The enzymatic activity was calculated according to Equation (S1).

$$A = \frac{\Delta a}{\Delta \mathrm{t}} \cdot \frac{V}{\varepsilon \cdot d \cdot v} \tag{S1}$$

Where, $A$ is the enzymatic volumetric activity in [U/mL], $a$ the absorbance, $t$ the time in [min], $V$ the volume in [mL], $\varepsilon$ the molar extinction coefficient in [mL/mol · cm] with 9700 mL/mol · cm, $d$ the cuvette path length in [cm] and $v$ the enzyme volume in [µL].

**Estimation of $CO_2$ footprint of phosphate buffer components**

Since no direct life cycle inventory data for $KH_2PO_4$ and $K_2HPO_4$ was available, the footprint was estimated via stoichiometric back-calculation. The product-related $CO_2$ footprint was calculated based on the stoichiometric reaction of the synthesis and the $CO_2$ footprints of the educts, that were available at ecoinvent.org. For the synthesis of $K_2HPO_4$ following reaction was used:

$$H_3PO_4 + 2\,KCl \rightarrow K_2HPO_4 + 2\,HCl \tag{S2}$$

Supplementary Table S4. Lists the molecular masses, the $CO_2$ footprints obtained from ecoinvent.org v3.12 https://ecoquery.ecoinvent.org/3.12/cutoff (accessed January 12 2026), and the estimated $CO_2$ footprints of the compounds included in the reaction equation.

| Compound | Molecular mass | $CO_2$ footprint |
|---|---|---|
| $H_3PO_4$ | 0.09799 kg/mol | 1.34 kg $CO_2$/ kg eq |
| KCl | 0.07455 kg/mol | 0.66 kg $CO_2$/ kg eq |
| $K_2HPO_4$ | 0.17418 kg/mol | 0.93 kg $CO_2$/ kg eq (see below) |
| HCl | 0.03646 kg/mol | Not estimated |

1. $CO_2$ contributions of educts

$$CO_2, H_3PO_4 = 1.34 \cdot 0.09799 = 0.1313\, kg\, CO_2/\, mol \quad \text{(S3)}$$

$$CO_2, KCl = 0.66 \cdot (2 \cdot 0.07455) = 0.0983\, kg\, CO_2/\, mol \quad \text{(S4)}$$

2. Total $CO_2$ input

$$CO_2, total = 0.1313 + 0.0983 = 0.2296\, kg\, CO_2/\, mol\, reaction \quad \text{(S5)}$$

3. Product and coproduct mass balance

$$product\ mass\ (total) = (0.17418 \cdot 1) + (2 \cdot 0.03646) = 0.24710\, kg \quad \text{(S6)}$$

$$x\,(K_2HPO_4) = 0.17418/\, 0.24710 = 0.7049 \quad \text{(S7)}$$

4. Allocation of $CO_2$ emissions

$$CO_2, alloc = 0.2296 \cdot 0.7049 = 0.1618\, CO_2/mol\, K_2HPO_4 \quad \text{(S8)}$$

5. Normalization to product mass

$$CO_2, K_2HPO_4 = 0.1618/0.17418 = 0.93\, kg\, CO_2/\, K_2HPO_4 \quad \text{(S9)}$$

For the synthesis of $K_2HPO_4$ following reaction was considered:

$$H_3PO_4 + KOH \rightarrow KH_2PO_4 + H_2O \quad \text{(S10)}$$

Supplementary Table S5. Lists the molecular masses, the $CO_2$ footprints obtained from ecoinvent.org v.3.12 https://ecoquery.ecoinvent.org/3.12/cutoff (accessed on 12 January 2026), and the estimated $CO_2$ footprints of the compounds included in the reaction equation.

| Compound | Molar mass | $CO_2$ footprint |
|---|---|---|
| $H_3PO_4$ | 0.09799 kg/mol | 1.34 kg $CO_2$/ kg eq |
| KOH | 0.05611 kg/mol | 4.73 kg $CO_2$/ kg eq |
| $KH_2PO_4$ | 0.13609 kg/mol | 2.57 kg $CO_2$/ kg eq (as above) |
| $H_2O$ | 0.01802 kg/mol | Not estimated |

**Cultivation of *V. natriegens* in BioLector XT®**

To investigate the influence of pH regulating compounds on bacterial growth, cultures of *V. natriegens* were cultivated in minimal medium supplemented with 10 g/L glucose as the sole carbon source. For all experiments, precultures were grown overnight in LBvn medium (Supplementary Table S6) at 30 °C and 180 rpm. Prior to inoculation of the main cultures, cells were harvested and washed twice with carbon-source-free minimal medium. Main cultivations were performed in a BioLector XT® (Beckmann Coulter, USA) system using a microfluidic feed plate (Lot no.2401221) containing the respective acids and bases KOH 0.5 M (Carl Roth, Germany), HCl 0.5 M (Carl Roth, Germany), NaOH 0.5 M (Carl Roth, Germany), and $H_2SO_4$ 0.5 M (Carl Roth, Germany).

Supplementary Table S6. Lists the different components and their final concentrations in the minimal medium and LB medium used for *Vibrio natriegens* cultivation.

| Stock solution | Medium Compound for VN minimal medium | Final concentration in media (g/L) |
|---|---|---|
| 1 | $Na_2HPO_4$ (Carl Roth, Germany) | 12.71 |
| 1 | $KH_2PO_4$ (Carl Roth, Germany) | 1.17 |
| 1 | NaCl (Carl Roth, Germany) | 25.71 |
| 1 | $NH_4Cl$ (Carl Roth, Germany) | 1.18 |
| 2 | $MgSO_4 \cdot 7H_2O$ (Carl Roth, Germany) | 1.63 |
| 3 | $CaCl_2 \cdot 2H_2O$ (Carl Roth, Germany) | 0.0735 |
| 4 | EDTA $\cdot$ $2Na_2 \cdot 2H_2O$ (Carl Roth, Germany) | 0.05 |
| 4 | $FeCl_3 \cdot 6H_2O$ (Carl Roth, Germany) | 0.0083 |
| 4 | $ZnCl_2$ (Carl Roth, Germany) | 0.00084 |
| 4 | $CuCl_2 \cdot 2H_2O$ (Carl Roth, Germany) | 0.00013 |
| 4 | $CoCl_2 \cdot 2H_2O$ (Carl Roth, Germany) | 0.0001 |
| 4 | $H_3BO_3$ (Carl Roth, Germany) | 0.0001 |
| 4 | $MnCl_2 \cdot 4H_2O$ (Fluka, Switzerland) | 0.000016 |
| 5 | Glucose (Carl Roth, Germany) | 10 |
| **Stock solution** | **Medium Compound for LBvn** | **Final concentration in media (g/L)** |
| 1 | LB (Carl Roth, Germany) + 10 g/L NaCl (Carl Roth, Germany) | 20 (with 15 g/L NaCl) |

The cultures were inoculated to an initial optical density ($OD_{600}$) of 0.1 in 1 mL minimal medium containing 10 g/L glucose (Carl Roth, Germany). Cultivations were carried out for 48 h at 30 °C and 1200 rpm under aerobic conditions. During cultivation, backscatter, dissolved oxygen (DO), and pH were monitored online using the BioLector XT® (Beckmann Coulter, USA) system. After cultivation, 1 mL samples were collected for biomass determination. Samples were centrifuged at 13000 × g for 5 min, and the supernatant was discarded. Subsequently, the open reaction tubes were dried at 60 °C for one week. The resulting dry biomass was quantified gravimetrically using a QUINTIX35-1S (Sartorius Lab Instruments, Germany) balance. Supplementary Figure S1 shows the cultivation profiles over 48 h including all monitored channels.

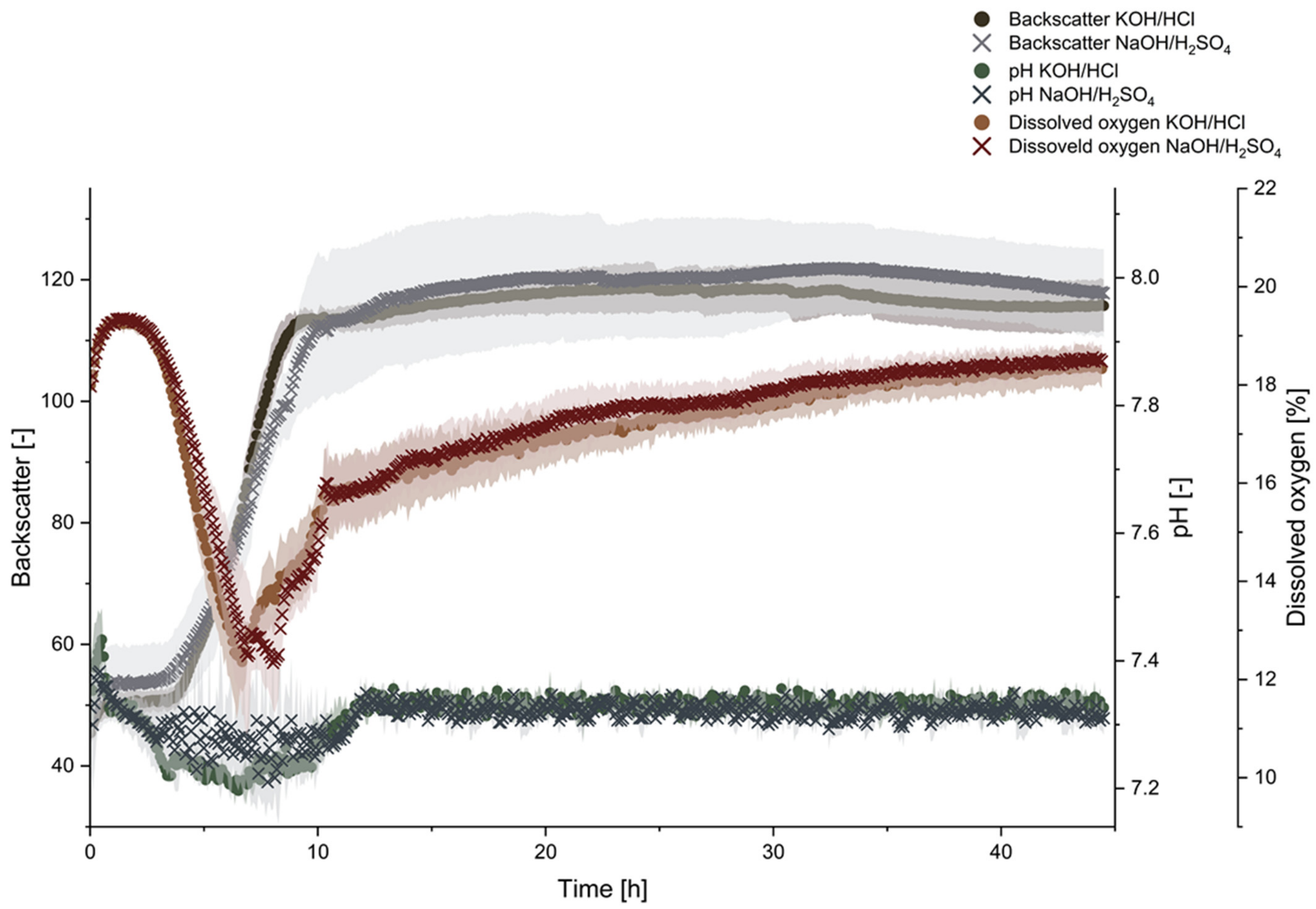


Supplementary Figure S1. Shows the BioLector XT® (Beckmann Coulter, CA, USA) cultivation profiles of *V. natriegens* grown in minimal medium containing 10 g/L glucose over 48 h, including all monitored channels (black: backscatter; green: pH; red: dissolved oxygen). Data points represent cultures with KOH/HCl-based pH regulation, while crosses indicate cultures regulated using $NaOH/H_2SO_4$.

**Cultivation of *M. extorquens* in BioLector XT® (Beckmann Coulter, CA, USA)**

An investigation was conducted into the discrepancy in $CO_2$ efficiency with the carbon sources methanol, succinic acid and ethanol being examined. The microorganism *M. extorquens* AM1 *Δcel* used in this study was kindly provided by the laboratory of Prof. Dr. Tobias Erb. The preculture was obtained from a cryo culture in a shaking flask containing media 1 (5 g/L peptone (Carl Roth, Germany), 3 g/L meat extract (Fluka, Switzerland) and 10 g/L methanol (Carl Roth, Germany)). It was incubated at 30 °C, 180 rpm for 68 h ($OD_{600} \approx 6.5$) before the culture was transferred into 50 mL falcons, centrifuged at 4 °C, 4816 x g for 5 min. The supernatant was discarded and the cell pellet washed with PBS (8 g/L NaCl (Carl Roth, Germany), 1.42 g/L $Na_2HPO_4$ (Carl Roth, Germany), 0.24 g/L $KH_2PO_4$ (Carl Roth, Germany) and 0.2 g/L KCl (Carl Roth, Germany)). The centrifugation was repeated and the $OD_{600}$ adjusted to 10 with PBS. The minimal media composition is shown in Supplementary Table S7.

The selection of buffer pH 6.7 (Supplementary 7 Stock solution 2) was contingent upon the carbon source, with succinic acid being utilized in conjunction with this buffer. Conversely, when methanol and ethanol were employed as the carbon source, buffer pH 7.1 (Supplementary 7 Stock solution 3) was employed. The carbon sources were used in concentrations of 30 mM and 100 mM and the wells were inoculated to an $OD_{600}$ of 1.

The cultivation on various carbon sources was conducted in a parallelized microbioreactor BioLector XT® (Beckmann Coulter, CA, USA) system. The assignment of the wells was randomized with the web-based application PlateDesigner (Suprun M, Suarez-Farinas M. PlateDesigner: a web-based application for the design of microplate experiments. Bioinformatics. 2018). The FlowerPlate® (Beckmann Coulter, CA, USA) (Lot no. 2506201) equipped with modules for measuring biomass, dissolved oxygen and pH was used in this study. Each condition was tested in triplicates in 1 mL

culture volume and the cultivation was performed at 30 °C, 1200 rpm shaking frequency and 10 mL/min air flow. At the start and the end of the experiment samples of the cultures were collected and centrifuged at 16900 x g for 2 min. The cell pellet was dried at 60 °C and used for cell dry weight determination. The supernatant was used for HPLC analysis using Agilent 1200 Series/1260 Infinity II high-performance liquid chromatography system combined with a Rezex™ ROA-organic acid H+ column (Phenomenex, CA, USA). The carbon source concentrations were detected through a refractive index detector at 50 °C or a variable wavelength detector. The column was operated at 40 °C with a flow rate of 0.5 mL/min 5 mM $H_2SO_4$ (Carl Roth, Germany) as eluent. The standards were measured at concentrations of 1 mM to 200 mM (600 mM for methanol (Carl Roth, Germany)) in eight concentration steps.

Supplementary Table S7. Lists the different components and their final concentrations in the minimal medium for *Methylorubrum extorquens*.

| **Stock solution** | **Medium Compound for minimal medium** | **Final concentration in media (g/L)** |
|---|---|---|
| 1 | $NH_4Cl$ (Carl Roth, Germany) | 1.62 |
| 1 | $MgSO_4 \cdot 7H_2O$ (Carl Roth, Germany) | 0.20 |
| 2 | $K_2HPO_4$ (Carl Roth, Germany) | 1.59 |
| 2 | $NaH_2PO_4 \cdot 2H_2O$ (Carl Roth, Germany) | 1.80 |
| 3 | $K_2HPO_4$ (Carl Roth, Germany) | 2.40 |
| 3 | $NaH_2PO_4 \cdot 2H_2O$ (Carl Roth, Germany) | 1.08 |
| 4 | $Na_2EDTA \cdot 2H_2O$ (Carl Roth, Germany) | 0.015 |
| 4 | $FeSO_4 \cdot 7H_2O$ (Carl Roth, Germany) | 0.003 |
| 5 | $ZnSO_4 \cdot 7H_2O$ (Carl Roth, Germany) | 0.0045 |
| 5 | $CoCl_2 \cdot 6H_2O$ (Carl Roth, Germany) | 0.003 |
| 5 | $MnCl_2$ (Sigma-Aldrich, MO, USA) | 0.00064 |
| 5 | $H_3BO_3$ (Carl Roth, Germany) | 0.001 |
| 5 | $Na_2MoO_4 \cdot 2H_2O$ (Sigma-Aldrich, MO, USA) | 0.0004 |
| 5 | $CuSO_4 \cdot 5H_2O$ (Riedel-de Haën, Germany) | 0.0003 |
| 5 | $CaCl_2 \cdot 2H_2O$ (Carl Roth, Germany) | 0.003 |
